# Electron microscopic tomography reveals discrete transcleft elements at excitatory and inhibitory synapses

**DOI:** 10.3389/fnsyn.2015.00009

**Published:** 2015-06-10

**Authors:** Brigit High, Andy A. Cole, Xiaobing Chen, Thomas S. Reese

**Affiliations:** ^1^Laboratory of Neurobiology, National Institute of Neurological Disorders and Strokes, National Institutes of HealthBethesda, MD, USA; ^2^Department of Cell and Molecular Biology, Northwestern UniversityChicago, IL, USA

**Keywords:** electron microscopy, EM tomography, synapses, synaptic cleft, adhesion molecules

## Abstract

Electron microscopy has revealed an abundance of material in the clefts of synapses in the mammalian brain, and the biochemical and functional characteristics of proteins occupying synaptic clefts are well documented. However, the detailed spatial organization of the proteins in the synaptic clefts remains unclear. Electron microscope tomography provides a way to delineate and map the proteins spanning the synaptic cleft because freeze substitution preserves molecular details with sufficient contrast to visualize individual cleft proteins. Segmentation and rendering of electron dense material connected across the cleft reveals discrete structural elements that are readily classified into five types at excitatory synapses and four types at inhibitory synapses. Some transcleft elements resemble shapes and sizes of known proteins and could represent single dimers traversing the cleft. Some of the types of cleft elements at inhibitory synapses roughly matched the structure and proportional frequency of cleft elements at excitatory synapses, but the patterns of deployments in the cleft are quite different. Transcleft elements at excitatory synapses were often evenly dispersed in clefts of uniform (18 nm) width but some types show preference for the center or edges of the cleft. Transcleft elements at inhibitory synapses typically were confined to a peripheral region of the cleft where it narrowed to only 6 nm wide. Transcleft elements in both excitatory and inhibitory synapses typically avoid places where synaptic vesicles attach to the presynaptic membrane. These results illustrate that elements spanning synaptic clefts at excitatory and inhibitory synapses consist of distinct structures arranged by type in a specific but different manner at excitatory and inhibitory synapses.

## Introduction

Synaptic formation, maintenance, and plasticity rely on coordination between multiple types of structural proteins spanning the synaptic cleft, the gap separating the pre- and postsynaptic membranes (Rees et al., 1976; Peters et al., 1991; Yamagata et al., 2003; Missler et al., 2012). These transcleft proteins comprise a diverse group of molecules, with the most proteins being members of the cadherin (Tepass et al., 2000) and immunoglobin (Rougon and Hobert, 2003) superfamilies. Other members include Eph receptors and their ephrin ligands (Kullander and Klein, 2002), neuroligins, and laminins such as neurexins and netrins (Graf et al., 2004). The structural diversity of transcleft molecules is matched by the diversity of their functions. For example, cadherin signaling regulates the recruitment of synaptic vesicles to the presynaptic active zone (Gottmann, 2008) while EphrinB promotes the recruitment of glutamate receptors and postsynaptic scaffolding proteins to the synapse (Waites et al., 2005). Neurexin/neuroligin complexes play an important role in the maintenance of the optimal ratio of excitatory and inhibitory synapses (Yamagata et al., 2003; Levinson and El-Husseini, 2005; Dalva et al., 2007) and knock out of these molecules leads to profound deficits in synaptic transmission (Graf et al., 2004; Missler et al., 2012). Neurexin/neuroligin complexes also work in conjunction with N-cadherin in order to regulate synaptic density during the initial stages of synaptogenesis (Aiga et al., 2011).

Conventional thin-section electron microscopy reveals dense material filling the synaptic cleft (Gray, 1959) that is typically bisected by a dense line, thought to represent a network of filamentous structures to anchor the pre- and postsynaptic membranes (Hajós, 1980; Landis and Reese, 1983; Ichimura and Hashimoto, 1988; Rostaing et al., 2006; Burette et al., 2012). The clefts of excitatory synapses maintain a gap of ~20 nm between the pre and postsynaptic membranes (Palay, 1956), the latter displaying small structures projecting from intramembrane particles (Landis and Reese, 1983). Some clefts remain intact even in isolated synaptosomes (Cotman, 1974), indicating that the structures bridging the cleft are necessary for maintaining the gap width of the cleft (Missler et al., 2012). The clefts of inhibitory synapses maintain a smaller gap of ~12 nm (Gray, 1959). Inhibitory synapses manifest a much thinner layer of postsynaptic electron-dense material extending over a longer synaptic contact (Gray, 1959; Linsalata et al., 2014).

Transcleft proteins directly attach to either the pre or postsynaptic membrane and pair across the cleft, forming either heterophilic or homophilic connections bridging the cleft. NCAM, N-cadherin, and SALMs typically bind homophilically across the cleft while SynCAMs, neurexin, neuroligin, ephrinB, EphB receptor, NetrinG, and NetrinG ligands typically bind heterophilically (Yamagata et al., 2003; Dalva et al., 2007; Fogel et al., 2007). The same cleft molecules may have different binding partners at excitatory and inhibitory synapses. For example, neurexin binds leucine-rich repeat transmembrane proteins (LRRTMs) at excitatory synapses (Linhoff et al., 2009) and binds GABA receptors, dystroglycan and neurexophilin at inhibitory synapses (Petrenko et al., 1996; Missler et al., 2012). Myriad spliced isoforms of transcleft molecules are selectively expressed in excitatory and inhibitory synapses (Tabuchi and Südhof, 2002), for example, neuroligin1 is localized exclusively at excitatory synapses (Song et al., 1999) while neuroligin2 is localized exclusively at inhibitory synapses (Varoqueaux et al., 2004). Neuroligins 3 and 4 are expressed in both types of synapse (Graf et al., 2004). SynCAM proteins also undergo alternative splicing to produce isoforms (Biederer, 2006) and bind in the specific, heterophilic complexes, SynCAM1 and 2 or SynCAM3 and 4 (Fogel et al., 2007).

Here, using transmission electron microscope (TEM) tomography, we delineate the structure and distribution of elements bridging the synaptic cleft at excitatory and inhibitory synapses in disassociated hippocampal cultures. High-pressure freezing and freeze substitution combined with EM tomography enables resolution of individual molecules in the synaptic cleft (Chen et al., 2008b; Linsalata et al., 2014). Although previous work using frozen sections of synapses revealed large complexes in synaptic clefts by cryo-EM and cryo-TEM tomography (Lucić et al., 2005; Zuber et al., 2005), individual cleft elements were not well resolved due to the low contrast of cryo-EM images. The present work presents a first look at the arrangements of individual proteins bridging the synaptic clefts at excitatory and inhibitory synapses.

## Materials and Methods

### Cultured Hippocampal Neurons

Dissociated rat hippocampal neurons were plated onto glia layers and grown for 20 days in culture in 10% CO_2_ in the 3-mm diameter gold specimen chamber designed for high-pressure freezing. All the synapses examined here were segmented and rendered from tomograms (Chen et al., 2008a,b, 2011) prepared to examine the postsynaptic densities at excitatory (Chen et al., 2011) and inhibitory synapses (Linsalata et al., 2014). The methods are summarized below but more detail can be found in Chen et al. (2008b) as well as in the publications cited above.

### Freeze Substitution

Cultures were covered with hexadecane and then frozen at 2100 Bar with a Bal-Tec HPM 010 machine in 124 mM NaCl, 2 mM KCl, 1.24 mM KH_2_PO_4_, 1.3 mM MgCl_2_, 2.5 mM CaCl_2_, 30 mM glucose, 25 mM HEPES, and 0.5% ovalbumin at a pH 7.4 and osmolarity of 325. They were then placed on frozen, saturated uranyl acetate and 2% acrolein in HPLC-grade acetone at −160°C for 15 min in an AFS Leica unit, ramped from −160 to −90°C over 14 h, held at −90°C for 8 h, ramped to −60°C during 6 h, and then held for 12 h. Samples were infiltrated in Lowicryl HM20 resin in acetone, and polymerized by UV at −50°C. Sections ~100–200 nm thick were cut *en face* and mounted on Formvar/carbon-coated grids. 10 nm gold particles were adhered to both sides of a grid as fiducial markers for fine alignment of the images to build a tomogram.

### Tomography

Excitatory and inhibitory synapses were distinguished and selected in sections prior to collecting tomographic series. Identification of inhibitory synapses was based on a group of structural characteristics (Linsalata et al., 2014). Inhibitory synapses exhibited more extensive sites of synaptic contact, thinner postsynaptic accumulations of electron-dense material, patchy discontinuities in this electron-dense material, and were located exclusively on dendritic shafts and somas. Synapses lacking ice crystal damage were imaged with FEI Tecnai 300-kV electron microscope equipped with field-emission gun at a dose of ~1000 electrons per nm^2^. Series were acquired in two orthogonal axes at tilt increments of 2° from +70° to −70° at pixel sizes of 0.48–0.75 nm to produce 2048 × 2048 images. Tomograms were aligned in two axes using IMOD.

### Analysis of Tomograms

Electron dense material spanning the cleft was segmented using Amira (FEI) in serial virtual sections ~1.4 nm thick calculated from tomograms. Two clefts each from excitatory and inhibitory synapses from two experiments were segmented and rendered, selecting only elements connected across the entire cleft in the sampled areas. Ten examples of each type of transcleft element, five from each of two experiments, were selected for measurements to determine the average dimensions of their filamentous elements using the 3D measurement function in Amira. The middle, upper, and lower diameters as well as the cleft angle of the transcleft elements were measured to find their mean thickness throughout their length and their degree of incline toward the postsynaptic membrane. Each type of element was also counted to sample its abundance within the cleft. For purposes of measurement, the length of the cleft was divided into equal sized peripheral and central zones, defining the peripheral zone as the two outermost quartiles of the cleft area in the tomogram and the center as the two innermost quartiles. The abundance of each type of transcleft element in each zone was calculated to determine which cleft element types were concentrated in the central or periphery of the cleft. Type-specific measurements included the ratio of the heights of type A cleft elements’ central bulges from the presynaptic membrane and from the postsynaptic membrane to determine bias in the central bulge’s position as well as the extent of presynaptic attachment in type D cleft elements.

The mean cleft width was measured with the 3D measurement function in Amira—five measurements from the central region and five from both peripheral regions in experiments 1 and 2. Cleft width was determined as the separation between the outer limit of the presynaptic membrane and the outer portion of the postsynaptic membrane. The area of cleft occupied by cleft elements was also calculated, with each type of cleft element extrapolated as a cylindrical object. These measurements are expressed with one standard deviation from the mean of the data.

## Results

**Figure 1:**
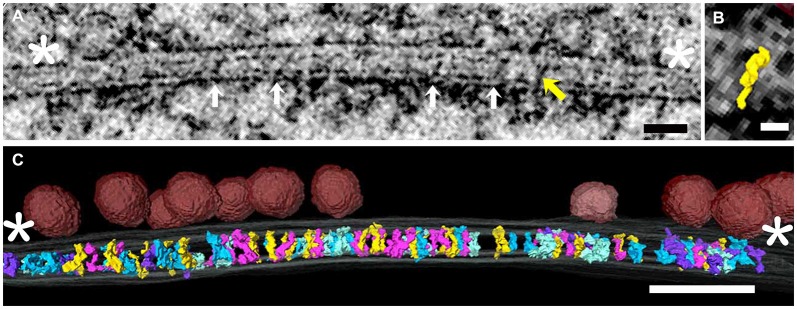
**(A)** Virtual section through an excitatory synapse, including both pre- and postsynaptic elements and elements spanning the synaptic cleft. This is one of 80–100 serial virtual sections derived from the tomogram, so not every element in the tomogram is evident in every section. Cleft elements (white arrows in successive virtual section were segmented one by one). The yellow oblique arrow indicates one structure that happened to cross the cleft in this virtual section. The cleft was considered to end at the white asterisks, and only elements crossing the cleft were rendered. **(B)** Surface rendering of the single element in the synaptic cleft indicated by yellow arrow in **(A)**. **(C)** Three dimensional rendering of all elements spanning the cleft. Various colors code the different structural types of cleft element. The same color-coding is used in Figure 2. Docked vesicles in the presynaptic active zone are in red. Scale bars: **(A)** 50 nm; **(B)** 10 nm; **(C)** 50 nm. Virtual section in **(A)** is 1.4 nm thick.

Tomograms were made from four freeze-substituted synapses, two excitatory and two inhibitory. These were identified in 100–120 nm thick sections based on criteria previously established for freeze-substituted hippocampal cultures (Chen et al., 2008a; Linsalata et al., 2014). The contrast achieved with freeze-substitution allowed differentiation of structures at molecular levels of detail in virtual sections from tomograms. Objects in 1.4 nm virtual sections were viewed at full resolution and readily segmented in all three orthogonal axes throughout the series of virtual sections. All elements spanning the cleft, considered to be the region between red arrows in Figure 1 and defined by constrictions in the intercellular space (Landis and Reese, 1983), were segmented and included in the data (Figure 1). Those that did not cross the cleft were typically attached to the either the pre or postsynaptic membrane.

### Transcleft Elements at Excitatory Synapses

**Figure 2:**
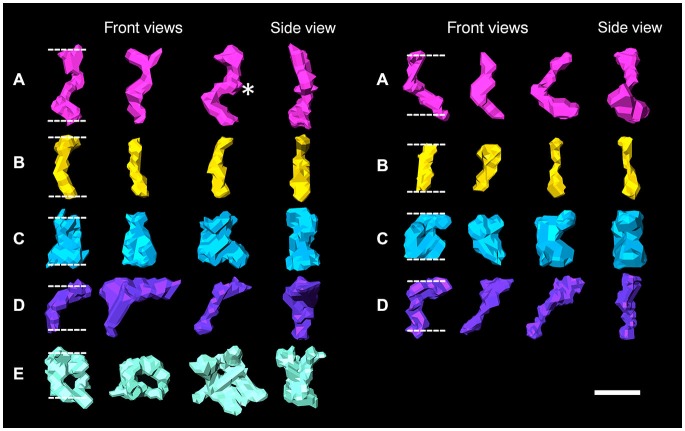
**Surface renderings of individual elements bridging the synaptic cleft are color-coded according to size and shape in both an excitatory synapse (left) and an inhibitory synapse (right)**. Synaptic cleft elements at excitatory synapses fall into five categories: **(A)** S-shaped elements, asterisk indicates bulge (pink), **(B)** straight elements (yellow), **(C)** rook-shaped elements (blue), **(D)** sickle-shaped elements (purple), **(E)** doughnut-shaped elements, third indicates an element with three stalks (turquoise). Elements at inhibitory synapses fell into similar categories except the first category **(A)** has C-shaped elements and no elements fall into the fifth **(E)** category. Scale bar: 10 nm.

Transcleft elements were readily classified based on their shape and dimensions. Five types of cleft elements bridged the synaptic cleft (Figure 2; Table 1). All transcleft elements were contained within the span (15.5 ± 2.4 nm, experiment 1, 17.2 ± 3.2 nm in experiment 2) of the synaptic cleft. Transcleft elements occasionally formed lateral contacts with each other, typically midway across the cleft (Figure 5). Cleft elements were typically inclined off the vertical axis by 15°–30°.

**Table 1:** **Distribution of elements in the synaptic cleft of excitatory synapses by type**.

	Type A	Type B	Type C	Type D	Type E
*Number of elements*	55	66	40	29	18
*Frequency*	26.5%	33%	18.5%	14%	8%
Diameter of element shaft (nm)*	5.0 ± 1.1	4.1 ± 1.2	7.2 ± 1.7	4.2 ± 0.8	3.9 ± 1.2
Diameter of upper base (nm)*	4.7 ± 0.9	4.3 ± 0.9	8.1 ± 2.0	10.6 ± 2.3	4.0 ± 1.4
Diameter of lower base (post)*	4.5 ± 1.1	4.5 ± 1.4	8.0 ± 2.0	4.8 ± 1.6	4.4 ± 1.2
*Distribution*	Center	Center	Periphery	Periphery	Scattered
% of total at the periphery**	24%	34%	79%	80%	47%

*mean of middle, upper, and lower diameters from 10 elements.**periphery defined as the outer 50% area of the cleft.

**Figure 3:**
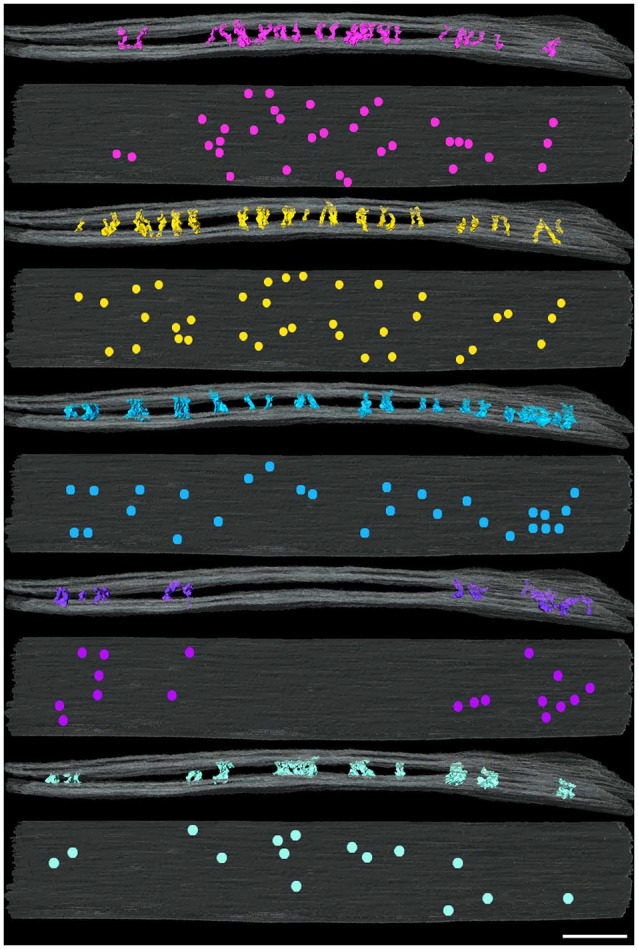
**Distribution of cleft elements by type at an excitatory synapse**. Side (above) and *en face* (below) views of synaptic clefts from an excitatory synapse in which each pair of views illustrates the localization for only one of the five types of elements coded as in Figure 2. Each of the five types of elements manifests different distributions in the cleft. Scale bar: 50 nm.

Type A and B elements had the smallest shaft diameters among the cleft elements. Type A elements were S-shaped with a shaft 4.7 ± 1.0 nm in diameter that manifested a bulge about a third of the distance from the post to the presynaptic membrane. Type B cleft elements were relatively straight compared with type A cleft elements, did not exhibit a medial bulge, and were 4.3 ± 1.1 nm in diameter throughout their shaft. Type A and B cleft elements comprise 27% and 33% of all transcleft elements respectively, and 76% of type A cleft elements and 66% of type B cleft elements reside in the central, rather than the peripheral domain, of the cleft (Figure 3).

Type C and D cleft elements had the largest diameters. In particular, type C elements manifested a thick stalk 7.8 ± 1.8 nm in diameter throughout their length. Type D elements showed less uniformity throughout their length, and were steeply curved, with shafts 4.4 ± 1.1 nm in diameter and enlarged feet 10.6 ± 2.3 nm in diameter contacting the presynaptic membrane. Type C and D elements comprised 19% and 14% of all transcleft elements, respectively, and 80% of both type resided in the peripheral domain of the cleft (Figure 3).

Type E elements appeared as complex, doughnut-shaped structures with central holes oriented parallel to the plane of the post-synaptic membrane. Some of the doughnuts were comprised of three or more stalks 4.3 ± 1.7 nm in diameter and two or more holes, suggestive of a complex of several interlocking curved filaments. Type E elements comprised 8% of all cleft elements and were distributed evenly throughout the synaptic cleft, with 47% residing in its periphery (Figure 3).

### Transcleft Elements at Inhibitory Synapses

**Figure 4:**
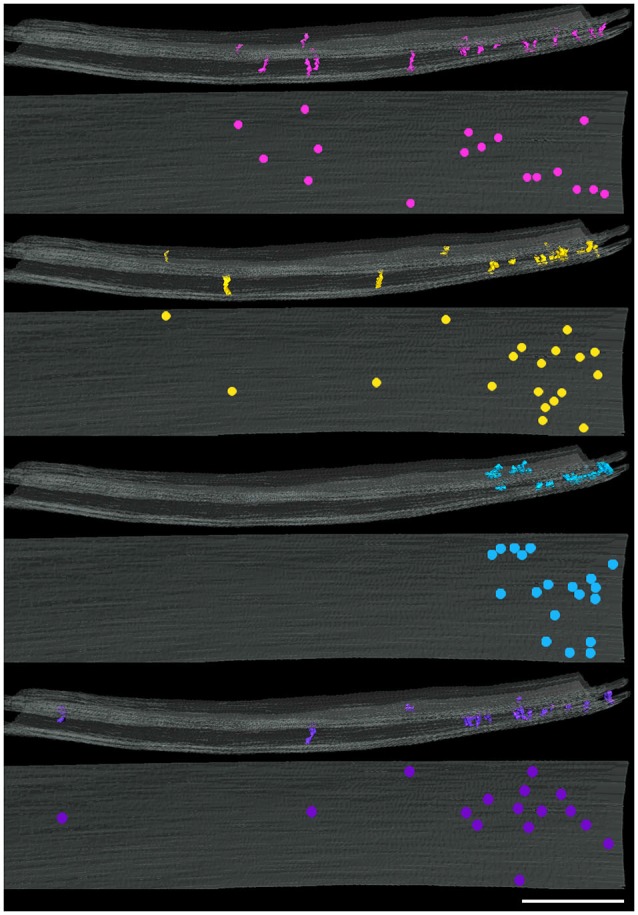
**Distribution of cleft elements by type at an inhibitory synapse**. Side (above) and *en face* (below) views of synaptic clefts from an inhibitory synapse in which each pair of views illustrates the localization for only one of the four types of elements coded as in Figure 2. Each of the four types of elements present in the cleft manifest similar distributions in a narrowed region of the periphery of the cleft. Scale bar: 50 nm.

Four types of transcleft elements were distinguished in the synaptic clefts from two inhibitory synapses (Figure 2). Even though the clefts at the inhibitory synapses are narrower overall, 10.1 ± 2.8 nm vs. 16.9 ± 2.9 nm, the shapes and proportional frequencies of the transcleft elements resembled four of the five classes (A–D) found at excitatory synapses. The organization of elements in the clefts at inhibitory synapses differed markedly from those at excitatory synapses. It became apparent in the tomograms that clefts at inhibitory synapses were as narrow as 6 nm in certain areas. The narrowed areas occupied up to a quarter of the cleft area near the periphery of the synapse. A total of 72% of transcleft elements were confined to the narrowed area of clefts situated near the periphery of the cleft (Figure 4). Transcleft elements were typically inclined from the vertical between 15° and 35° with respect to the postsynaptic membrane and displayed more inclination in the narrower parts of the cleft. Transcleft elements fitting within the cleft where it narrowed to 6 nm also appeared to be shorter than those in wider parts (Figure 2).

Cleft elements in inhibitory clefts, which resembled type A cleft elements in excitatory synapses, formed C-shaped rather than S-shaped structures and comprised 25% of all transcleft elements in inhibitory synapses. They were 2.4 ± 0.6 nm in diameter and had a single bulge along their shafts about halfway between the pre- and postsynaptic membrane (Figure 2; Table 2). These transcleft elements were the most uniformly distributed of the cleft elements, with only 54% located in the narrowed part of the cleft (Figure 4). Cleft elements resembling type B elements in excitatory synapses had a straight shaft 2.8 ± 0.5 nm in diameter (Figure 2; Table 2). They were the most numerous of the four types of cleft elements in inhibitory synapses, comprising a third of all the elements and they typically appeared in the narrowed part of the cleft, where 67% resided (Figure 4).

**Table 2:** **Distribution of elements in the synaptic cleft of inhibitory synapses by type**.

	Type A	Type B	Type C	Type D
*Number of elements*	38	37	22	25
*Frequency*	32%	30%	18%	20%
Diameter of element shaft (nm)*	2.3 ± 0.8	2.7 ± 0.6	4.9 ± 0.8	2.5 ± 0.6
Diameter of upper base (nm)*	2.4 ± 0.5	2.7 ± 0.4	4.8 ± 1.4	2.6 ± 0.6
Diameter of lower base (post)*	2.4 ± 0.7	2.9 ± 0.5	5.1 ± 1.4	2.6 ± 0.6
*Distribution*	Polar right	Polar right	Right edge	Polar right
% of total at the periphery**	54%	67%	96%	77%

*mean of middle, upper, and lower diameters from 10 elements.**periphery corresponds to area of narrowed cleft width.

Cleft elements resembling type C elements had a thick, stalk-like structure similar to their counterparts at excitatory synapses. The diameter of their shafts (5 ± 1.2 nm) was the largest among the cleft elements at inhibitory synapses (Table 2). Type C elements appeared truncated at the narrowed region of the cleft where 96% resided (Figures 2, 4) and comprised 20% of all the elements at the inhibitory synapse. Cleft elements classified as type D were 2.5 ± 0.6 nm in diameter and made elongated contacts 7.5 ± 1.8 nm in diameter with the presynaptic membrane (Table 2). These elements closely matched their possible counterparts at excitatory synapses (Figure 2). Type D transcleft elements at inhibitory synapses comprised 20% of all the elements in the cleft and 77% of these resided in the narrowed domain of the cleft (Figure 4).

### Distribution of Transcleft Elements in the Vicinity of Docked Synaptic Vesicles

**Figure 5:**
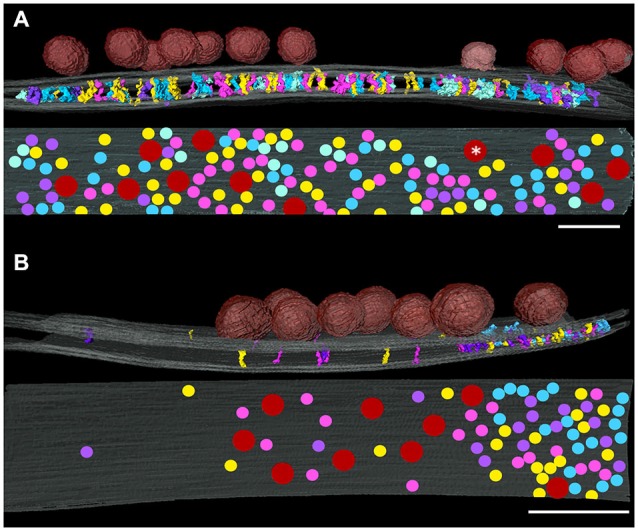
**Relationship of the distribution of synaptic vesicles at an excitatory (A) and an inhibitory synapse (B) projected onto presynaptic membrane (gray) as red disks**. The white asterisk indicates a vesicle appearing to be fusing on the presynaptic membrane. Contacts of cleft elements with the presynaptic membrane appear to avoid synaptic vesicle contacts. Scale bar: 50 nm.

In order to investigate relationships between transcleft elements and synaptic vesicle docking sites, maps of the distributions of transcleft elements in excitatory and inhibitory synapses were overlaid with renderings of synaptic vesicles attached to the presynaptic membrane. In the clefts of both excitatory and inhibitory synapses, transcleft elements were absent under attached synaptic vesicles (Figure 5), including below a vesicle appearing to be fusing with the presynaptic membrane (Figure 5A). While docked vesicles in excitatory synapses were distributed evenly over the whole synaptic cleft, those in inhibitory synapses were concentrated in the central domain of the cleft (Figure 5B) and did not form attachments to the portion of the presynaptic membrane participating in the narrowed area of the cleft with the denser distribution of transcleft elements.

## Discussion

Electron microscope tomography of intact hippocampal culture synapses reveals five types of discrete elements crossing the synaptic cleft at excitatory synapses. This picture differs from images derived from cryoelectron microscopy of synaptic clefts in organotypic hippocampal slices where some cleft elements appear regularly spaced 8.2 nm apart when analyzed at resolution of 5–10 nm (Zuber et al., 2005). A second study uses synaptosomes from rat neocortex to combine tomography with cryoelectron microscopy to characterize the structures in the synaptic cleft. Segmentation of the virtual sections at a pixel size of 2.2 nm reveals large transcleft complexes irregularly spaced and occupying 10% of the cleft volume (Lucić et al., 2005). Variation in cleft structure might be expected between synapses formed by different classes of neurons.

Transcleft elements in the present study are analyzed in virtual sections from tomograms derived from freeze-substituted cultures, resolving individual elements as small as 2–5 nm. Preparing synapses for EM tomography by freeze-substitution has the advantage over cryoelectron microscopy that the heavy metal stain introduced during freeze-substitution provides images with a high signal-to-noise ratio, resulting in clear definition of individual protein segments in virtual sections of tomograms (Chen et al., 2008a, b). EM images were collected near focus for generating tomograms with 1.4 nm voxels from which cleft elements were manually segmented for surface rendering. At this level of definition, it is apparent that the cleft is bridged by numerous individual structures that may make lateral contact but generally remain separated from each other. In contrast to the large molecular complexes apparent with cryoelectron microscopy, these discrete cleft elements occupy approximately five percent of the total cleft volume in excitatory synapses. There is a lack of any apparent barriers to diffusion in any direction in the cleft, consistent with observations that synaptic clefts are essentially open for small molecules to diffuse through (Brightman and Reese, 1969).

At least a dozen species of molecules extend across the synaptic cleft (Missler et al., 2012). In contrast, only five types of transcleft elements can be distinguished on the basis of their shapes, distributions, and dimensions, suggesting that some types of transcleft elements could represent more than one species of transcleft molecule or that some cleft elements are beyond detected with the freeze-substitution methods. Indeed, sequence comparisons of neuroligins 1, 3, and 4 suggest their structures could be too similar to distinguish with freeze-substitution (Ichtchenko et al., 1996; Südhof, 2008). Because cleft elements extend completely across the cleft from membrane-to-membrane without apparent extension beyond the cleft, we presume that the majority of cleft elements represent the ectodomains of transcleft proteins. The level of detail in the cleft structures uncovered by tomography suggests that the question of whether cleft elements represent protein species with specific molecular identities can ultimately be tested by measuring fits of different type of cleft elements to crystal structures.

The neurexin/neuroligin complex is an example of a possible candidate for a match with one of the types of transcleft element. The ectodomains of neurexin1β and neuroligin1 form heterotetramers that crystallize into a lateral, sheet-like structure under physiological conditions (Tanaka et al., 2012), but heterotetramerized neurexin1β and neuroligin4 does not form a lateral sheet (Leone et al., 2010). The crystallized ectodomains of neurexin1β/neuroligin1 complexes are ~12 nm high by ~8 nm wide at a resolution of ~3Å  (Tanaka et al., 2012). A space-filling structure of the neurexin1β/neuroligin1 complex exhibits similar dimensions to that of the 5 nm shaft of type A cleft elements as well as their characteristic S-shape.

The cadherins, netrins, and ephrins might also be represented among the types of transcleft elements. The ectodomains of N-cadherin (Harrison et al., 2011) form dimers with *cis* and *trans* interactions ~26 nm high by 7 nm wide. The crystal structure of the *cis* N-cadherin dimer exhibits the straight shape and a shaft 4 nm in diameter typical of filamentous type B cleft elements. The ectodomains of complexes of NetrinG2 and NetrinG2 ligand are ~16 nm in height by ~8 nm wide at a resolution of ~2Å  (Seiradake et al., 2011). Type C cleft elements are ~8 nm wide and resemble the trapezoidal shape of the space-filling crystal structure of NetrinG2 and NetrinG2 ligand in complex. EphrinB and EphB receptor form tetrameric, ring-like complexes (Himanen et al., 2001). Although the dimensions of its crystal structure were not reported, the crystal structure of EphrinB and EphB complex exhibits the characteristic doughnut shape of type E transcleft elements). High-resolution structures are not reported for other transcleft molecules.

Identifications of transcleft element types can be inferred by comparing known distributions of transcleft molecules with the distributions of the five cleft element types. For example, immunogold labeling has shown that N-cadherin and protocadherin adhesion systems are located at the periphery of the cleft (Uchida et al., 1996) where cleft element types C and D typically reside. N-cadherin, recruited early during synaptogenesis, mediates initial axo-dendritic adhesion (Benson and Tanaka, 1998). Immunogold labeling of SALMs and neurexin/neuroligin complexes localizes these molecules throughout the cleft (Song et al., 1999; Seabold et al., 2008) along with neurexin, neuroligin, SynCAM, EphrinB and EphB receptor (Waites et al., 2005).

No transcleft elements lie under docked vesicles suggesting a strategic role for the organization of transcleft elements in synaptic vesicle release and recycling. Involvement of several transcleft molecules in modulation of the synaptic vesicle cycle during long-term potentiation has been posited (Gottmann, 2008). Knockdown of N-cadherin results in reduced mini-EPSC frequency, and knockout of NCAM prevents paired-pulse facilitation and increases synaptic depression, implying impaired mobilization or docking of vesicles (Gottmann, 2008). Molecular identification of transcleft elements in excitatory synapses might provide further information about their role in synaptic transmission.

The transcleft elements at inhibitory synapses, like those at excitatory synapses, appear as discrete structures in tomograms prepared by freeze-substitution. These transcleft elements are readily classified into four types roughly corresponding to four of the types at excitatory synapses despite the narrower synaptic clefts. Some of these shorter transcleft elements might represent truncated versions of the corresponding elements in excitatory synapses, because many classes of transcleft molecules engage in alternative splicing and express multiple isoforms (Missler et al., 2012). Alternatively, some elements appeared more tilted, which would accommodate them in the narrower cleft. Several species of transcleft molecule are known to exist at inhibitory synapses, including neurexin, neuroligin-2, SALM and NCAM (Yamagata et al., 2003; Graf et al., 2004; Varoqueaux et al., 2004; Mah et al., 2010). However, except for neuroligin2 (Varoqueaux et al., 2004), detailed localizations of most synaptic cleft molecules are not yet available.

The transcleft elements at inhibitory synapses exhibit structural and organizational features distinct from those at excitatory synapses. The width of the cleft at inhibitory synapses is 10 nm as compared to 16 nm at excitatory synapses. Indeed, clefts of inhibitory synapse typically exhibit elongated, discontinuous postsynaptic specializations and thinner cleft widths (Linsalata et al., 2014). Clefts of inhibitory synapses also exhibit a further narrowing to ~6 nm wide in zones at the periphery of the cleft, and most of the transcleft element types are confined to these zones.

Final molecular identification of the elements in the synaptic cleft will require additional approaches. It may be useful to examine the transcleft elements after acute knockdown of different species of transcleft molecules, as any deletions of entire types of transcleft elements could reveal an obvious pattern. Immunolabeling could also be used to verify the location of different types of transcleft molecules, while mass spectrometry analysis could determine copy numbers of each type of transcleft molecule. Through the combination of these techniques, it should be possible to identify each of the types of structures present in the clefts of excitatory and inhibitory synapses and provide definitive information about their numbers and distributions. These data will help clarify how the different proteins bridging the synaptic clefts at excitatory and inhibitory synapses maintain their multiple functions (Missler et al., 2012) and regulate synaptic transmission.

## Conflict of Interest Statement

The authors declare that the research was conducted in the absence of any commercial or financial relationships that could be construed as a potential conflict of interest.
